# MiR-9 Promotes Angiogenesis via Targeting on Sphingosine-1- Phosphate Receptor 1

**DOI:** 10.3389/fcell.2020.00755

**Published:** 2020-08-07

**Authors:** Xinghong Yao, Linshen Xie, Ye Zeng

**Affiliations:** ^1^Institute of Biomedical Engineering, West China School of Basic Medical Sciences and Forensic Medicine, Sichuan University, Chengdu, China; ^2^Department of Radiation Oncology, Sichuan Cancer Hospital & Institute, School of Medicine of University of Electronic Science and Technology of China, Chengdu, China; ^3^West China School of Public Health and West China Fourth Hospital, Sichuan University, Chengdu, China

**Keywords:** sphingosine-1-phosphate receptor, miR-9, angiogenesis, endothelial cell, microRNA

## Abstract

We previously demonstrated that vascular endothelial cells released VEGF-enriched exosomes to promote the tumor vasculogenesis and progression after anti-angiogenic therapies (AATs). To clarify how microRNA (miR)-9 promoted the angiogenesis of tumor-associated endothelial cells, in the present study, we investigated the association between miR-9 and sphingosine-1-phosphate (S1P) receptors in angiogenesis. The levels of miR-9 and S1P receptors in normal and tumor endothelial cells were compared with EndoDB database and their correlations were analyzed. The levels of S1P_1_, S1P_2_, and S1P_3_ were detected in miR-9 overexpressing endothelial cells by qRT-PCR and western blot. The binding sites of miR-9 on S1P_1_ and S1P_3_ were predicted and tested by dual-luciferase reporter assays. Then, angiogenesis in endothelial cells overexpressing both S1P_1_ and miR-9 was detected. The results showed that miR-9 is overexpressed in ECs from medulloblastoma and glioblastoma xenograft, which is negatively associated with S1P_1_ and S1P_3_. Overexpression of miR-9 significantly inhibited S1P_1_ and S1P_3_ in both mRNA and protein levels. We predicted that binding sites exist between miR-9 and S1P_1_, S1P_3_, but only S1P_1_ was directly targeted by miR-9. Overexpression of S1P_1_ significantly suppressed the miR-9-induced angiogenesis. Therefore, miR-9 induces angiogenesis via targeting on S1P_1_.

## Introduction

Angiogenesis is an important progress during physiological and pathophysiological development. Angiogenesis is a complex process of vessel growth but in the strictest sense denotes vessels sprouting from pre-existing ones (Potente et al., 2011). Inadequate angiogenesis causes ischemia in myocardial infarction, stroke, and neurodegenerative or obesity-associated disorders, whereas excessive angiogenesis promotes many ailments including cancer, inflammatory disorders such as atherosclerosis, and eye diseases. Anti-angiogenic therapies (AATs) have been developed to combat tumor metastasis (Hosein et al., 2020). Nowadays, AATs strategies include blood vessel pruning, disruption or normalization of the tumor vasculature, and tumor immunosensitization but did not yield satisfactory efficacy (Cully, 2017; Cloughesy et al., 2020). Bevacizumab antagonizes vascular endothelial growth factor (VEGF) to induce vascular normalization and therefore reduce edema. Vascular disruptive agents such as VB-111 disrupts the angiogenic vasculature via promoting tumor starvation and enhancing the vascular permeability in the tumor environment to increase edema and recruit the immune cells (Cloughesy et al., 2020). AATs and immune checkpoint inhibitors were combined to acquire promising outcomes of cancer patients (Yi et al., 2019). However, how and by which mechanism does the intratumoral vessel form remains unclear.

The angiogenetic process is rather complex involving localized breakdown of the basement membrane and extracellular matrix of a pre-existing vessel, proliferation, and migration of capillary endothelial cells (ECs) into the surrounding tissue, and new vessel formation. Stimulating by the proangiogenic signals such as hypoxia (Cantelmo et al., 2017) and cytokines (e.g., VEGF; Gerhardt et al., 2003), ECs become motile and invasive. MicroRNAs (miRNAs) have recently been shown to regulate gene expression associated with tumorigenesis and angiogenesis (Zhuang et al., 2012; Zeng et al., 2019). Oncogenic miR-9 is significantly elevated in breast cancer cells (Ma et al., 2010), hepatocellular carcinoma (Zhuang et al., 2012; Drakaki et al., 2015), squamous cell carcinomas (White et al., 2013), lung and colorectal carcinoma (Ma et al., 2010), and ovarian cancer (Laios et al., 2008). MiR-9 is significantly increased in ECs upon *in vitro* tumor-ECs co-cultures. We recently demonstrated that miR-9 promotes angiogenesis via activating the autophagy pathway (Zeng et al., 2019). MiR-9 also promotes the angiogenesis of endothelial progenitor cells via activating the autophagy pathway (Zhou et al., 2020). However, the molecular mechanism involved in miR-9-induced angiogenesis in ECs has not been fully explored.

G protein-coupled sphingosine-1-phosphate (S1P) receptors including S1P_1_ (formerly endothelial differentiation gene-1, EDG-1), S1P_2_ (EDG-5), and S1P_3_ (EDG-3) are abundant in ECs. During vascular development *in vivo*, S1P_1_ is required in ECs (Gaengel et al., 2012). Decrease of S1P_1_ increases angiogenic sprouting and destabilization of the endothelium, while the activation of S1P_1_ signaling inhibits angiogenic sprouting and enhances cell-to-cell adhesion (Gaengel et al., 2012; Cartier and Hla, 2019). It was reported that S1P_2_ inhibited tumor angiogenesis in ECs in mouse models (Du et al., 2010). S1P_3_ promotes EC migration and plays a critical role in developmental and pathological angiogenesis (Jin et al., 2018). Therefore, we hypothesis that miR-9 induces angiogenesis in ECs via targeting S1P receptors.

In the present study, we aimed to investigate which S1P receptor is regulated by miR-9 and aberrant expressed in ECs during angiogenesis. The miR-9 was transfected into the ECs to simulate the tumor-associated ECs as previously described (Zeng et al., 2019). The mRNA and protein levels of S1P_1_, S1P_2_, and S1P_3_ were detected. Then, miR-9 binding sites of the S1P receptors were predicted and verified by Dual-Luciferase Reporter Assay. The role of putative S1P receptor in cell migration, invasion, and angiogenesis was explored. A rescue assay was performed to validate that the putative S1P receptor is a bona fide antigenic target regulated by miR-9 in ECs.

## Materials and Methods

### Expression of miR-9 and S1P Receptors in Normal ECs and Tumor ECs

Databases E-GEOD-73753 [normal hindbrain ECs, *n* = 8; Sonic hedgehog (Shh)-driven medulloblastoma ECs, *n* = 8; Wnt-driven medulloblastoma ECs, *n* = 8], E-MTAB-3949 (normal ECs, *n* = 2; angiogenic glioblastoma xenograft ECs, *n* = 2), GSE-77199 [colorectal cancer ECs (colon), *n* = 4; colon ECs, *n* = 3; ECs from colorectal metastasis to the liver, *n* = 4; liver ECs, *n* = 4; renal cell carcinoma ECs, *n* = 4; renal ECs, *n* = 4], E-GEOD-51401 (CD105+ ECs from hepatocellular carcinoma, *n* = 7; CD105+ normal ECs, *n* = 8; CD31+ ECs from hepatocellular carcinoma, *n* = 8; CD31+ normal ECs, *n* = 8) and E-GEOD-41614 (vessels isolated from bladder cancer tissues, *n* = 5; vessels isolated from bladder normal tissues, *n* = 5) in EndoDB^1^ from Khan et al. (2019) were obtained. In the human genome, three independent miR-9 loci including miR-9-1, miR-9-2, miR-9-3 reside at chromosomes 1, 5, and 15, respectively, which share an identical mature miR-9 sequence. The expression of miR-9-1, miR-9-2, miR-9-3, S1P_1_, S1P_2_, and S1P_3_ in those normal ECs and tumor ECs were compared. The relationships between the significant differentially expressed miR-9 loci and specific S1P receptors were analyzed.

### Cell Culture

HUVECs (Allcells, Shanghai, China) were cultured in HUVEC medium (HUVEC-004; Allcells) as previously described (Zeng et al., 2019). Cells (passages three to six) were maintained at 37°C with 5% CO_2_ in a humidified incubator. Lentivirus infections of miR-9 mimic (5′-TCTTTGGTTATCTAGCTGTATGA-3′) and negative control (NC; 5′-UUGUACUACACAAAAGUACUG-3′; B04001; GenePharma, Shanghai, China) were performed as previously described (Zeng et al., 2019). cDNAs for S1P_1_ were ligated into plasmid pcDNA3.1. For overexpression of S1P_1_, cells were transfected with pcDNA3.1 vector expressing S1P_1_ using Lipofectamine 2000 reagent (Invitrogen, Thermo Fisher Scientific, Waltham, MA, United States). Transfected cells after 48 h were used for the subsequent experiments. Recombinant human VEGF 165 Protein (50 ng/mL; hVEGF; 293-VE, R&D system, Minneapolis, MN, United States) was used to test the migration, invasion and *in vitro* angiogenesis of cells overexpressing S1P_1_.

### Real-Time Quantitative PCR Analysis

RNA was extracted from cells using TRIzol (Invitrogen) and qRT-PCR assays were performed using a SYBR Premix Ex Taq kit (TaKaRa, Shiga, Japan). The primer sequences were as follows: *S1P*_1_, 5′-TTTCTGCGGGAAGGGAGTATGT-3′ (forward) and 5′- GCAGGAAGAGGCGGAAGTTATT-3′ (reverse); *S1P*_2_, 5′-CTGTATGGCAGCGACAAGAGC-3′ (forward) and 5′-GAGGCAGGACAGTGGAGCAG-3′ (reverse); *S1P*_3_, 5′-TACGCACGCATCTACTTCCTGG-3′ (forward) and 5′-GCTCCGAGTTGTTGTGGTTGG-3′ (reverse); and *GAPDH*, 5′-TGTTCGTCATGGGTGTGAAC-3′ (forward) and 5′-ATGGCATGGACTGTGGTCAT-3′ (reverse). All primers and probes were obtained from TaKaRa. Gene expression was normalized to that of GAPDH using the 2^–△△CT^ method, and data are presented as expression relative to the indicated controls. The stem-loop primers and probes for mature miR-9 and U6 small nuclear (sn)RNA were as follows: hsa-miR-9-5p, 5′-ACACTCCAGCTGGGTCTTTGGTTATCTAG-3′ (forward) and 5′-CTCAACTGGTGTCGTGGAGTCGGCAATTCAGTTGAGT CATACAG-3′ (reverse); and U6 snRNA 5′-CTCGCTTCGGCAGCACA-3′ (forward) and 5′-AACGCTTCACGAATTTGCGT-3′ (reverse). The relative expression level of miR-9 was normalized to that of U6 snRNA and shown as a ratio relative to the expression level in the control. Data are representative of three independent experiments.

### Western Blot Analysis

Proteins were extracted from cells using radioimmunoprecipitation assay lysis buffer containing a protease inhibitor cocktail (Beyotime, Beijing, China). After determination of protein concentration using a protein determination kit (Cayman Chemical Company, Ann Arbor, MI, United States), equal amounts (20–30 μg) of protein samples were size-fractionated by sodium dodecyl sulfate-polyacrylamide gel electrophoresis, electrotransferred onto a polyvinylidene fluoride membrane (Millipore, United States), blocked with 5% non-fat milk in PBS, and hybridized with antibodies against S1P_1_ (TA311878; Origene, Rockville, MD, United States), S1P_2_ (TA311287; Origene), and S1P_3_ (TA321693; Origene) at 4°C overnight. A 1:1000 dilution of each antibody was used for detection. Glyceraldehyde-3-phosphate dehydrogenase (GAPDH) was used as an internal control. The blots were incubated with the corresponding horseradish peroxidase (HRP)-conjugated secondary antibodies (1:5000; Beyotime), and enhanced chemiluminescence was performed using an Immobilon Western Chemiluminescent HRP Substrate (WBKLS0050; Millipore, Billerica, MA, United States) to visualize the bands. Densitometric quantification was performed using ImageJ software (version 1.52u; National Institutes of Health, Bethesda, MD, United States).

### Dual-Luciferase Reporter Assay

The wild-type (WT) and the corresponding mutation (MUT) sites at 3′-untranslated regions (3′UTRs) of S1P_1_ and S1P_3_ were cloned into the psiCHECK-2 vector (Promega, Madison, WI, United States), respectively. The recombinant reporter plasmids were validated by DNA sequencing and then transfected into the miR-9 overexpressing cells and NC cells using Lipofectamine 2000 reagent. Luciferase activities were measured using the Dual-Luciferase Reporter Assay System (E1910, Promega). Renilla luciferase activity was normalized to Firefly luciferase activity.

### *In vitro* Angiogenesis Assay

The *in vitro* angiogenesis assay was performed as previously described (Zeng et al., 2019). The 24-well plates were coated with Matrigel (300 μL/well; BD Biosciences, San Jose, CA, United States) without introducing air bubbles. After gelling of the Matrigel, 5 × 10^4^ cells were plated into each Matrigel-coated well along with 200 μL of HUVEC basal medium containing 10% FBS. After 6 h incubation with or without 50 ng/mL hVEGF at 37°C with 5% CO_2_ in a humidified incubator, the medium was gently aspirated from each well and incubated with Diff-Quick fixative (Dade Behring, Deerfield, IL, United States) for 30 s and subsequently stained with solution II for 2 min. Tube structures were observed and imaged by microscopy. The pseudo-vascular organization of cells was analyzed by ImageJ software (version 1.52u) using the Angiogenesis Analyzer plugin^2^.

### Cell Migration and Invasion Assay

Cells were digested with 0.25% EDTA trypsin and resuspended in HUVEC basal medium (HUVEC-004B; Allcells) with or without 50 ng/mL hVEGF. Cells were seeded at a density of 1 × 10^5^ cells per Transwell (BD Biosciences), and HUVEC basal medium containing 10% fetal bovine serum (FBS; Hyclone; GE Healthcare, Logan, UT, United States) was added to the lower chamber. The Transwell membrane was precoated with Matrigel for invasion and not precoated for migration. After 48 h, cells that had migrated or invaded through the membrane were quantified as previously described (Zeng et al., 2019).

### Statistical Analysis

Statistical significance was determined by Student’s *t*-test or one-way analysis of variance with either the least significant difference test or Tamhane’s T2 test (depending on Levene’s statistic for homogeneity of variance) using SPSS software (v25.0; IBM, Armonk, NY, United States). The relationships between miR-9 and S1P receptors were measured using Pearson correlation methods. Data were presented as Mean ± SEM. *P* < 0.05 was considered statistically significant.

## Results

### MiR-9 Is Overexpressed in Tumor ECs, Which Is Negatively Associated With S1P_1_

**FIGURE 1:**
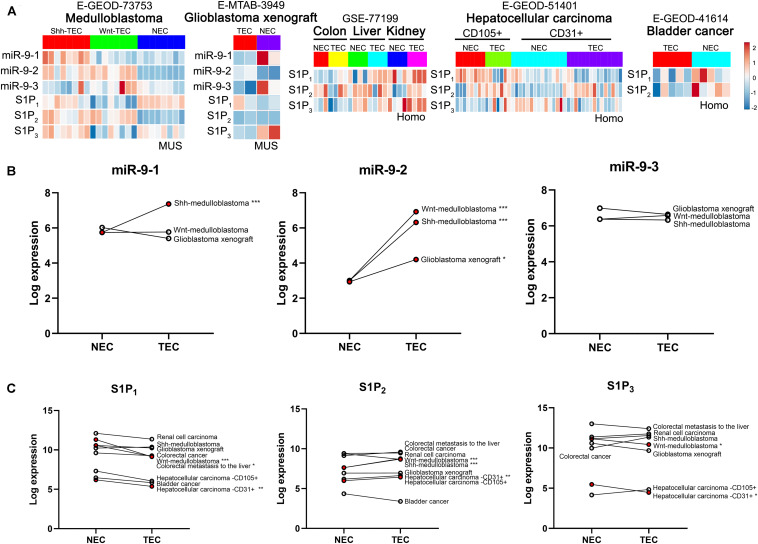
Expression analysis of miR-9 and S1P receptors in tumor ECs and normal ECs basing on EndoDB databases. **(A)** Gene expression heatmap of normal ECs (NEC) vs. tumor ECs (TEC). Red indicates high-expression levels, and blue indicates low-expression levels. **(B)** Expression of the miR-9-1, miR-9-2, and miR-9-3 in NECs and TECs. MUS: musculus; Homo: Homo sapiens. *P < 0.05, **P < 0.01, ***P < 0.001.

**FIGURE 2:**
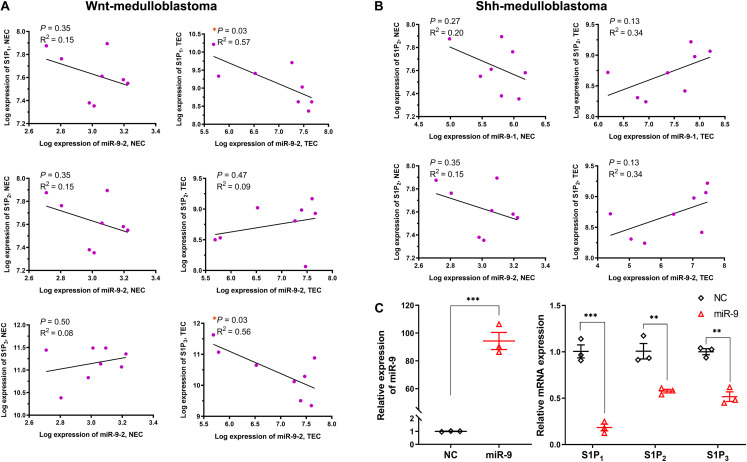
Relationship between miR-9 and S1P receptors. The correlation between the significant differentially expressed miR-9 loci and specific S1P receptors in normal ECs (NEC) and tumor ECs (TEC) were performed. **(A)** Correlation between miR-9-2 and S1P receptors in Wnt-medulloblastoma; **(B)** correlation between miR-9-1, miR-9-2, and S1P_2_ in Shh-medulloblastoma. Then, the levels of S1P receptors in HUVECs upon miR-9 mimics transfection were detected by qRT-PCR. **(C)** miR-9 levels in HUVECs overexpressing miR-9 (left) and mRNA expression of S1P_1_, S1P_2_, and S1P_3_ (right). NC: miR-9 mimics negative control. Mean ± SEM. *n* = 4, *P < 0.05, **P < 0.01, ***P < 0.001.

The levels of miR-9 and S1P receptors in normal ECs and tumor ECs in multiple tumors were compared (Figure 1). As shown in Figure 1A, databases E-GEOD-73753 and E-MTAB-3949 show miR-9-1, miR-9-2., miR-9-3, S1P_1_, S1P_2_, and S1P_3_ levels in Shh-medulloblastoma ECs, Wnt-medulloblastoma ECs, and ECs from angiogenic glioblastoma xenograft. Databases GSE-77199, E-GEDO-51401, and E-GEOD-41614 show levels of S1P receptors in colorectal cancer ECs, renal cell carcinoma ECs, hepatocellular carcinoma ECs, and bladder cancer ECs. Specifically, miR-9-1 was significantly upregulated in Shh-medulloblastoma ECs (Figure 2B). miR-9-2 was significantly upregulated in Wnt-medulloblastoma ECs, Shh-medulloblastoma ECs, and ECs from glioblastoma xenograft. miR-9-3 was not significantly changed between normal ECs and those tumor ECs. S1P_1_ was significantly downregulated in Wnt-medulloblastoma ECs, ECs from colorectal metastasis to liver, and hepatocellular carcinoma ECs (Figure 2C). S1P_2_ was significantly upregulated in medulloblastoma and hepatocellular carcinoma ECs. S1P_3_ was significantly downregulated in Wnt-medulloblastoma and hepatocellular carcinoma ECs. Taken together, miR-9 was significantly upregulated in multiple tumor ECs. It was accompanied by significant downregulation of S1P_1_ and S1P_3_.

Furthermore, the correlations between miR-9-2 and S1P_1_, S1P_2_, and S1P_3_ in normal ECs and medulloblastoma ECs were analyzed (Figures 2A,B). Results have shown miR-9-2 negatively correlated to S1P_1_ and S1P_3_ in Shh-medulloblastoma ECs, but there was no significant correlation in normal ECs. There was no significant correlation between miR-9-2 and S1P_2_ in both Shh- and Wnt-medulloblastoma ECs (Figures 2A,B). Thus, the upregulation of miR-9 in tumor ECs is significantly associated with the downregulation of S1P_1_ and S1P_3_.

### MiR-9 Negatively Regulates S1P_1_ and S1P_3_

**FIGURE 3:**
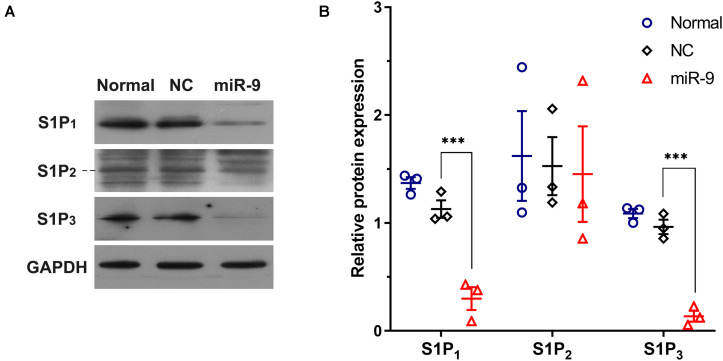
Western blot detection of S1P_1_, S1P_2_, and S1P_3_ in HUVECs overexpressing miR-9. Cell lysates were collected from HUVECs with or without NC or miR-9 transfection, and then 20 μg of protein samples were subjected to western blot analysis. **(A)** Western blot of cell lysates using anti-S1P1, anti-S1P2, and anti-S1P3. **(B)** The quantification was performed with ImageJ. The protein expression was normalized to the GAPDH level. NC: miR-9 mimics negative control. Mean ± SEM. *n* = 3, ***P < 0.001.

Upregulation of miR-9 in tumor-associated ECs was simulated by the transfection of miR-9-expressing lentivirus into HUVECs. A 94.3-fold increase in miR-9 was shown in the ECs overexpressing miR-9, compared with NC (Figure 2C). The mRNA levels of S1P_1_, S1P_2_ and S1P_3_ in the ECs overexpressing miR-9 were significantly downregulated (0.18 ± 0.04 vs. 1.01 ± 0.07 for S1P_1_, *P* < 0.001; 0.58 ± 0.02 vs. 1.01 ± 0.02 for S1P_2_, *P* < 0.01; 0.52 ± 0.05 vs. 1.00 ± 0.03 for S1P_3_, *P* < 0.01, Figure 2C). Levels of S1P_1_, S1P_2_, and S1P_3_ in the ECs overexpressing miR-9 were also detected by Western Blot assay (Figures 3A,B). There were no significant changes in levels of S1P_1_, S1P_2_, and S1P_3_ between normal and NC cells, while miR-9 overexpression significantly downregulated the levels of S1P_1_ (0.30 ± 0.11 vs. 1.20 ± 0.07, miR-9 vs. NC, *P* < 0.001) and S1P_3_ (0.13 ± 0.05 vs. 0.96 ± 0.07, miR-9 vs. NC, *P* < 0.001) in the ECs. The expression of S1P_2_ was not significantly changed in HUVECs overexpressing miR-9 (1.45 ± 0.44 vs. 1.53 ± 0.27, miR-9 vs. NC, *P* > 0.05). These results suggest that miR-9 significantly inhibited S1P_1_ and S1P_3_ in both mRNA and protein levels.

### MiR-9 Directly Downregulates the S1P_1_ but Does Not Directly Target the S1P_3_

**FIGURE 4:**
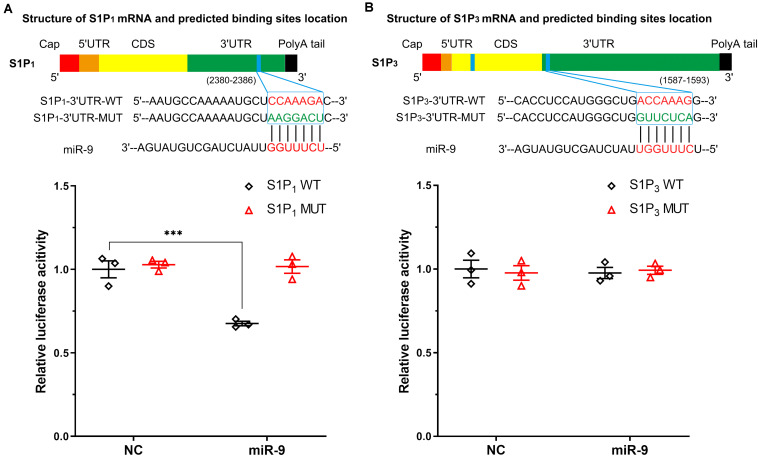
Associations between S1P_1_, S1P_3_, and miR-9 were detected by dual-luciferase reporter assay. The structures of S1P_1_ mRNA (transcript variant 1 M_001400.5, and transcript variant 2 NM_001320730.1; the variant 2 differs in the 5′UTR compared to variant 1, both variants 1 and 2 encode the same protein) and S1P_3_ mRNA (NM_005226.4) including coding DNA sequence (CDS) and untranslated regions were depicted. The blue band indicated the putative miR-9 binding sequence in the target mRNA molecules. The binding sites located at 3′UTR were screened for Dual-Luciferase Reporter Assay analysis. **(A)** Putative wild-type (WT) binding sequence and its mutation (MUT) sequence in the 3′UTR of S1P_1_. Luciferase activity was measured. **(B)** Putative WT binding sequence and its MUT sequence in the 3′UTR of S1P_3_. Luciferase activity was measured. NC: miR-9 mimics negative control. Mean ± SEM. *n* = 3, ***P < 0.001.

The binding sites between S1P_1_, S1P_3_, and miR-9 were predicted using TargetScan (Release 7.1^3^, Whitehead Institute for Biomedical Research, Cambridge, MA, United States) and microRNA.org (Release August 2010^4^, the Computational Biology Center at Memorial Sloan-Kettering Cancer Center, Rockville, MD, United States). There was a predicted miR-9 binding site (7mer-A1) at position 2380–2386 of S1P_1_ 3′UTR and two predicted miR-9 binding sites at position 1587–1593 (7mer-m8) of S1P_3_ 3′UTR and 816-822 (7mer-A1) (Figures 4A,B). However, the miR-9 binding site at 816-822 is within the coding region of S1P_3_ (NM_005226.4). Therefore, the luciferase reporter plasmids containing the WT and the corresponding MUT sites at 3′UTR of S1P_1_ (position 2380–2386) and S1P_3_ (position 1587–1593) were generated. The luciferase activity of cells with WT-3′UTR of S1P_1_ was significantly inhibited by miR-9, while the luciferase activity of cells with MUT-3′UTR of S1P_1_ was not significantly inhibited by miR-9 (Figure 4A). It was not observed a significant decrease in luciferase activity of cells with WT-3′UTR of S1P_3_ by miR-9, indicating that miR-9 was not directly targeted the 3′UTR of S1P_3_ (Figure 4B). These results suggest that S1P_1_ but not S1P_3_ is a bona fide miR-9 target.

### Overexpression of S1P_1_ Inhibits the VEGF-Induced Angiogenesis

**FIGURE 5:**
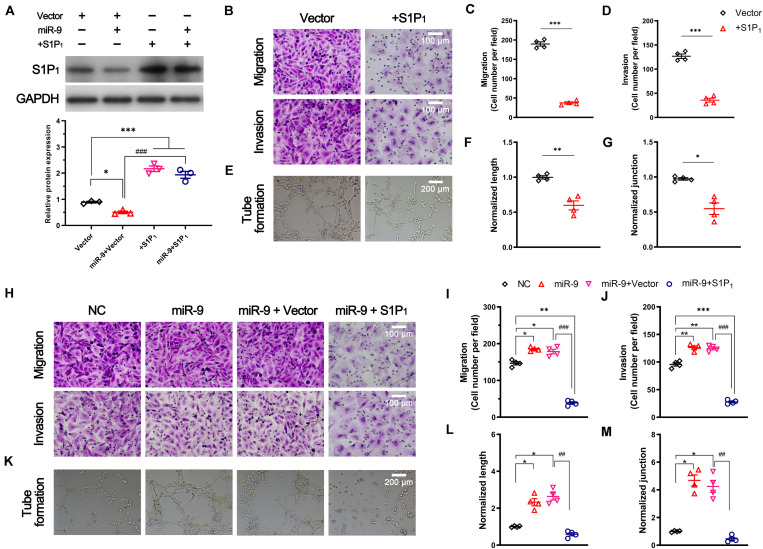
Effect of S1P_1_ on angiogenesis. Cells were transfected with pcDNA3.1 vector expressing S1P_1_ (+S1P_1_) or pcDNA3.1 vector (vector). For the rescue assay, cells overexpressing miR-9 were transfected with pcDNA3.1 vector expressing S1P_1_ (miR-9 + S1P_1_) or pcDNA3.1 vector (miR-9 + vector). After transfection for 48 h, the protein level, migration, invasion and *in vitro* angiogenesis were performed. **(A)** Western blot of S1P_1_ and quantification. Mean ± SEM. *n* = 3, *P < 0.05, ***P < 0.001. ^###^*P* < 0.001. **(B–D)** Transwell assays **(B)** of migration (top) and invasion (bottom) of HUVECs overexpressing S1P_1_ in response to 50 ng/mL hVEGF. Mean ± SEM, *n* = 4. ***P < 0.001. **(E–G)** Tube formation **(E)**, normalized tube length **(F)**, and number of junctions **(J)** formed by HUVECs overexpressing S1P_1_ in response to 50 ng/mL hVEGF. Mean ± SEM, *n* = 4. *P < 0.05, **P < 0.01. **(H–J)** Transwell assays **(H)** of migration (top) and invasion (bottom) of HUVECs overexpressing miR-9 and S1P_1_. NC: miR-9 mimics negative control. Mean ± SEM, *n* = 4. *P < 0.05, **P < 0.01, ***P < 0.001; ^###^*P* < 0.001. **(K–M)** Tube formation **(K)**, normalized tube length **(L)**, and the number of junctions **(M)** formed by HUVECs overexpressing miR-9 and S1P_1_. NC: miR-9 mimics negative control. Mean ± SEM, *n* = 4. *P < 0.05; ^##^*P* < 0.01.

To investigate the role of S1P_1_ in angiogenesis, S1P_1_ was significantly overexpressed in ECs (2.17 ± 0.11 vs. 0.89 ± 0.03, S1P_1_ overexpression vs. vector, *P* < 0.05, Figure 5A). Overexpression of S1P_1_ significantly suppressed 50 ng/mL VEGF-induced migration and invasion (Figures 5B–D). As a consequence, the overexpression of S1P_1_ significantly inhibited the VEGF-induced tube formation. The tube length and number of junctions in the network were significantly reduced by S1P_1_ overexpression (Figures 5E–G). These results suggest that overexpression of S1P_1_ inhibits the VEGF-induced angiogenesis in HUVECs.

### MiR-9 Promotes Angiogenesis via Downregulation of S1P_1_

We then investigated the role of S1P_1_ in miR-9-induced angiogenesis. A rescue assay demonstrated the significant upregulation of S1P_1_ in ECs overexpressing miR-9 (1.94 ± 0.23 vs. 0.50 ± 0.08, miR-9 + S1P_1_ vs. miR-9 + vector, *P* < 0.001, Figure 5A). The restoration of S1P_1_ expression significantly reversed the miR-9-induced migration, invasion, and angiogenesis in HUVECs (Figures 5H–M). The tube formation, tube length, and the number of junctions in the network induced by miR-9 were significantly reduced by the restoration of S1P_1_ (Figures 5K–M). These results suggest that miR-9 promotes angiogenesis via direct downregulation of S1P_1_.

## Discussion

Angiogenesis is considered one of the critical pathophysiological events in multiple disorders including atherosclerotic plaque rupture (Perrotta et al., 2020), cancer, and so on (Hosein et al., 2020). MiR-9 is elevated in atherosclerosis and many cancers such as breast cancer, hepatocellular carcinoma, squamous cell carcinomas, lung and colorectal carcinoma, and ovarian cancer (Laios et al., 2008; Ma et al., 2010; Zhuang et al., 2012; White et al., 2013; Drakaki et al., 2015). Our previous study has shown miR-9 induced angiogenesis via activating of the autophagy pathway (Zeng et al., 2019). Our new findings in the present study demonstrate that upregulation of miR-9-2 locus in tumor ECs is significantly associated with downregulation of S1P_1_ and S1P_3_, and further validate that S1P_1_ acts as a bona fide target for miR-9, and the downregulation of S1P_1_ contributes to EC migration, invasion, and angiogenesis.

The role of miR-9 in angiogenesis is a promising therapy for many diseases. MiR-9 could promote migration, invasion, and angiogenesis of endothelial progenitor cells via downregulating transient receptor potential melastatin 7 (TRPM7) and activating PI3K/Akt/autophagy pathway, which might facilitate thrombi recanalization in deep vein thrombosis (Zhou et al., 2020). Translocation of miR-9 from bone marrow-derived mesenchymal stem cells into vascular ECs could induce angiogenesis via activating PI3K/AKT pathway to repair the severe acute pancreatitis (Qian et al., 2018). Moreover, miR-9 promotes angiogenesis via the downregulation of CXC chemokine receptor-4 (CXCR4) and inhibition of PI3K/AKT/mammalian target of rapamycin (mTOR) pathway in HUVECs, thereby suppressing the high glucose-induced injury in HUVECs (Yi and Gao, 2019). It is unclear why both activation and inhibition of PI3K/AKT regulated by miR-9, but both of them involved in promoting angiogenesis in HUVECs. PI3K/AKT/mTOR pathway is an important regulator of autophagy. We previously described that miR-9 activated autophagy in HUVECs and reviewed that miR-9 may induce autophagy via targeted suppression of FOXO1, CUL4A, CK1α, GSK3β, Notch2, cyclin D1, and MCPIP1 (Zeng et al., 2019). In the present study, we verified that miR-9 directly bound to 3′UTR of S1P_1_. The overexpression of S1P_1_ inhibited the VEGF-induced angiogenesis. A rescue assay also demonstrated that restoration of S1P_1_ inhibited the miR-9 induced angiogenesis. For the rescue assay, the transfection efficacy of the pcDNA3.1 vector in EC is more than 80%. After transfected with pcDNA3.1 vector, S1P_1_ expression in ECs was reduced less by miR-9 compared with that without pcDNA3.1 vector transfection, without significant difference (by 43.4 ± 7.7% miR-9/NC vs. 74.3 ± 8.9% miR-9 + vector/vector, Mean ± SEM, *P* > 0.05, Figures 2, 4), suggesting the change in endogenous S1P_1_ could be disregarded. The slight change in reduction of S1P_1_ might be caused by too many vectors transfection into ECs overexpressing miR-9 or miR-9 trapped somewhere else by other cellular elements such as long non-coding RNAs.

Excessive tumor vessels were present in mice with endothelial cell-specific knockout of S1P_1_ (Cartier et al., 2020). Loss of S1P_1_ induced angiogenesis might be associated with increased VEGFR2 activity (Gaengel et al., 2012). Inhibition of S1P_1_ enhanced VEGFR2 activation in murine ECs upon murine VEGF injection (Fischl et al., 2019). The CD34 labeled vessels tended to increase with S1P_1_ inhibition, but the combined inhibition of S1P_1_ and VEGF pathways reduced blood flow in tumor, increased tumor cell apoptosis, and inhibited the tumor CD34 positive vessels in clear cell renal cell carcinoma tumor models (Fischl et al., 2019). We previously demonstrated that activation of S1P_1_ protects ECs against glycocalyx shedding and promotes the glycocalyx synthesis via activating PI3K signaling (Zeng et al., 2014, 2015). The association among S1P_1_, PI3K/AKT/mTOR and autophagy will be investigated in the future. In atherosclerosis, miR-9 upregulation is associated with inhibition of intracellular lipid accumulation and macrophage foam cell formation (Shao et al., 2020). Whether miR-9 contributed to the angiogenesis in atherosclerotic plaque rupture remains to be clarified by *in vivo* experiments.

Many biological functions of S1P_1_ on the vasculature and its expression are regulated by S1P. S1P acts primarily as an extracellular signaling molecule activated G protein-coupled S1P receptors including S1P_1_, S1P_2_, and S1P_3_ in ECs. S1P_1_ is the main S1P receptor expressed by ECs (Panetti, 2002). S1P induces migration of various ECs via S1P_1_ and S1P_3_ but does not induce the migration of nonendothelial cells (Panetti, 2002). It was demonstrated that S1P induces angiogenesis only in the presence of low levels of VEGF, providing a major caveat as an angiogenic factor (Panetti, 2002). S1P/S1P_1_ signaling activation inhibited VEGF-induced angiogenic responses (Jozefczuk et al., 2020). More than 50% of S1P is carried by high-density lipoprotein (HDL). HDL promotes angiogenesis via S1P/S1P_3_ mediated VEGFR2 activation (Jin et al., 2018). In the present study, we found that S1P_3_ was downregulated by miR-9. However, we failed to validate the predicted miR-9 binding sites within S1P_3_ 3′UTR. There might be an unknown intermediate element such as a transcription factor in the miR-9/S1P_3_ pathway. In the future, it will be worth to find the intermediate element and test how is S1P_3_ regulated by miR-9 and associated with angiogenesis. S1P is significantly reduced in cardiovascular diseases (Jozefczuk et al., 2020), and significantly evaluated in hepatocellular carcinoma tissues (Zeng et al., 2016). The relationship between miR-9 and S1P in a special environment should be carefully considered. Although the S1P_2_ mRNA was downregulated by miR-9, the protein level of S1P_2_ was only slightly decreased without significant difference. This might be due to the poor sensitivity of the commercially available S1P_2_ antibody. Moreover, the miR-9 binding site was not predicted within the S1P_2_ 3′UTR using the TargetScan and microRNA.org.

It was believed that most known miRNA target sites (miRNA seed) have 7 nt Watson-Crick seed matches (seed matches) (Lewis et al., 2003). The complete UTR sequence is most critical for mRNA recognition. After changing one or more base pairings between miRNAs and mRNAs, the target UTRs would not identify by miRNAs. We mutated 6 nt and had not tested other constructs with different mutations for S1P_1_. Moreover, it was demonstrated that the miRNA response elements might locate within the luciferase coding region (Campos-Melo et al., 2014). We predicted whether miR-9 targeting the mRNA sequence of Renilla luciferase gene (hRluc) or firefly luciferase gene (hluc+) by using custom prediction tools of miRbase (Release 22.1^5^; managed by the Griffiths-Jones lab at the Faculty of Biology, Medicine and Health, University of Manchester, United Kingdom) and miRDB (Xiaowei Wang’s lab^6^ at the Department of Radiation Oncology, Washington University School of Medicine in St. Louis, United States) databases. The predicted miRNAs do not include miR-9, suggesting luciferase could not modify our results.

In HUVECs, an about 94-folds overexpression of miR-9 was achieved artificially. Nevertheless, the luminescence in the miR-9 cells with WT-3′UTR of S1P_1_ was decreased by only about 40%. Bioluminescence is a chemical process in which an enzyme such as luciferase breaks down a substrate such as luciferin and one of the by-products of this reaction is light. The transfection efficacy of the psiCHECK-2 vector and conversion efficiency of luciferin to oxyluciferin might limit the diminution of the luminescence. It was worth noting that the psiCHECK-2 vector contains both renilla and firefly luciferase genes. As Renilla transcript expressed with the 3′UTR sequence of interest gene, Renilla luciferase activity is used as a measure of the effect of the 3′UTR on transcript stability and translation efficiency. Firefly luciferase is used to normalize transfections and eliminates the need to transfect a second vector control. The transfection efficacy of the psiCHECK-2 vector was not detected in EC.

In summary, building on previous findings that miR-9 overexpression in HUVECs promotes angiogenesis, and AATs triggers VEGF-enriched exosomes to promote tumor vasculogenesis, we present data to show that S1P_1_ acts as a bona fide target for miR-9 to regulate the migration, invasion, and angiogenesis. The expressions of S1P_1_/S1P_2_/S1P_3_ between normal-associated ECs and tumor-associated ECs should be carried out by *in vivo* and *in vitro* experiments such as normal cells/tumor cells and ECs crosstalk assays in the future. The investigation on the molecular mechanism whereby miR-9 promotes angiogenesis in HUVECs might provide a critical cue to reveal the AATs triggered exosomes release and further to control the tumor vasculogenesis and progression following AATs.

## Data Availability Statement

All datasets generated for this study are included in the article.

## Author Contributions

YZ contributed to the conception and design of the work. YZ, XY, and LX performed the acquisition, analysis, and interpretation of data for the work. YZ and XY drafted the manuscript. All authors contributed to the article and approved the submitted version.

## Conflict of Interest

The authors declare that the research was conducted in the absence of any commercial or financial relationships that could be construed as a potential conflict of interest.

## Footnotes

1 https://endotheliomics.shinyapps.io/endodb/

2 https://imagej.nih.gov/ij/macros/toolsets/Angiogenesis%20Analyzer.txt

3 http://www.targetscan.org/

4 http://www.microrna.org/

5 http://www.mirbase.org

6 http://mirdb.org/
